# Neurocysticercosis: An Uncommon Cause of Acute Supratentorial Hydrocephalus

**DOI:** 10.5334/jbsr.2742

**Published:** 2022-04-29

**Authors:** Olaf De Weerdt, Bénédicte Dubois, Philippe Demaerel

**Affiliations:** 1 Department of Radiology, University Hospitals Leuven, BE; 2 Department of Neurology, University Hospitals Leuven, BE; 3 Department of Radiology, University Hospital KU Leuven, BE

**Keywords:** magnetic resonance imaging, Taenia solium, intraventricular, neurocysticercosis, hydrocephalus

## Abstract

We report a 29-year-old woman with acute supratentorial hydrocephalus due to intraventricular neurocysticercosis (NC). Aqueductal stenosis due to web formation and a free floating intraventricular cyst with scolex were pathognomonic and led to the diagnosis of NC. Worldwide, NC is the most important parasitic infection of the central nervous system but is very uncommon in non-endemic regions. Intraventricular abnormalities occur in approximately 30% of the patients. Magnetic resonance imaging (MRI) plays a crucial role in the diagnostic work-up and in guiding intervention.

**Teaching Point:** Brain magnetic resonance imaging in intraventricular neurocysticercosis is pathognomonic and essential in guiding treatment.

## Introduction

NC is the most common parasitic infection of the central nervous system caused by the larval stage of the pork tapeworm Taenia solium. Increasing globalization, travelling, and migration has triggered the spread of NC in non-endemic regions. The cysts can be found in the parenchyma, in the subarachnoid space and/or in the ventricles. Ventricular cysts can cause cerebrospinal fluid (CSF) flow obstruction and/or arachnoiditis [1].

## Case Report

A 29-year-old Nepalese woman, who has been living in Belgium for 14 years, presented to the emergency department with worsening headache. She described unilateral headaches since 10 days with nausea and vomiting, not responsive to analgesics.

The neurological examination was unremarkable. There was no evidence of papilledema.

**Figure 1:**
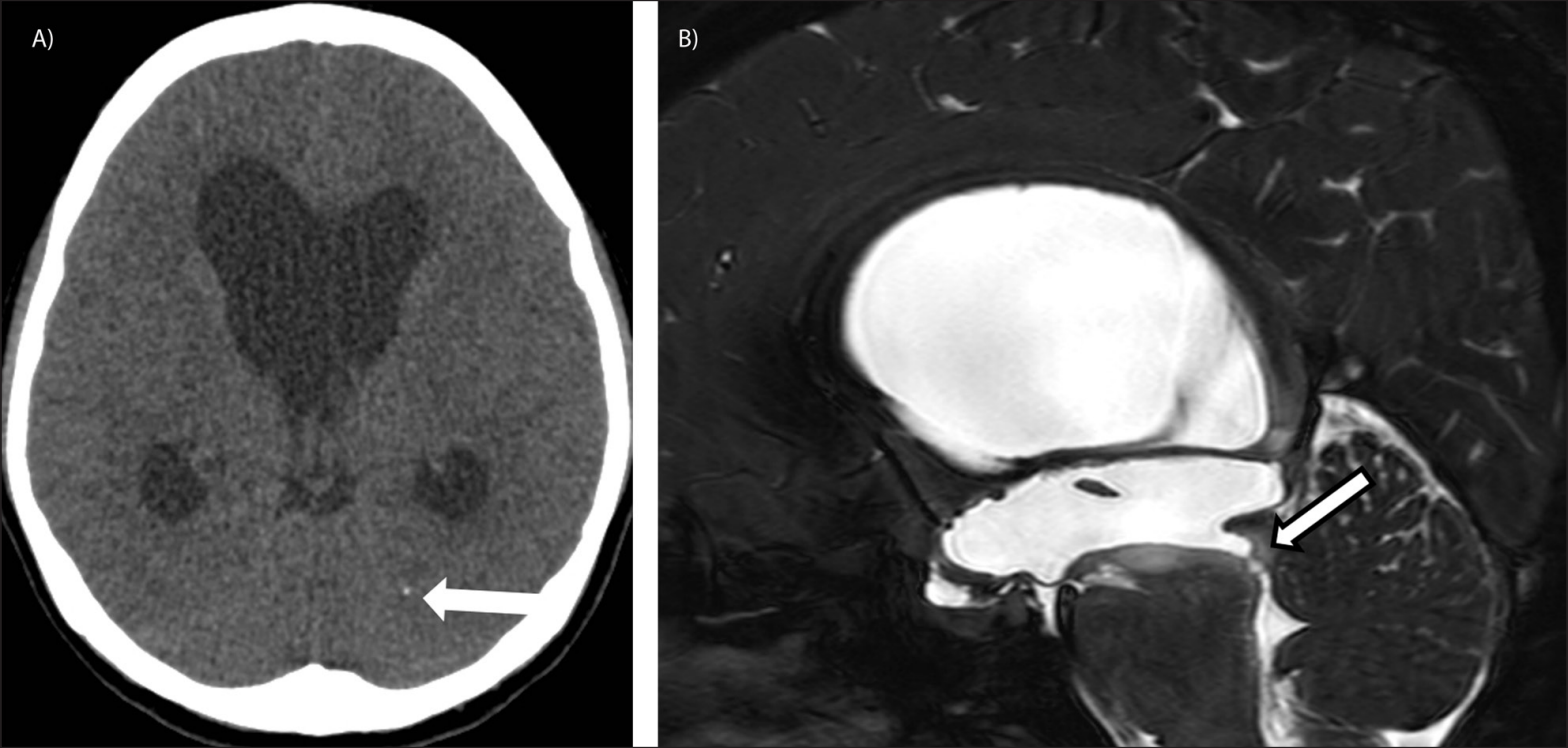
Brain CT on admission shows marked dilatation of the lateral ventricles and third ventricle. Note the calcification in the occipital lobe (**A**, arrow). The 3D CISS imaging-sequence demonstrates the aqueductal stenosis due to an intraluminal web (**B**, arrow).

**Figure 2:**
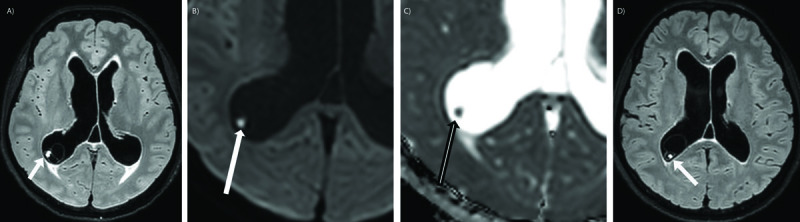
Brain MRI shows an intraventricular cyst with an eccentric nodule, representing the scolex (**A**, arrow). Note the diffusion restriction of the scolex (**B, C**, arrow). Following ventriculostomy the location of the floating cyst was slightly different (**D**, arrow).

Brain computed tomography (CT) showed dilatation of the supratentorial ventricles with signs of transependymal edema (***Figure 1a***). A parenchymal calcification in the left occipital region was noticed. Brain MRI confirmed the acute triventricular hydrocephalus with aqueductal stenosis due to an intraluminal web (***Figure 1b***). A lobulated cystic lesion was seen in the occipital horn of the right lateral ventricle containing a small solid eccentric nodule with diffusion restriction (***Figure 2a–c***). There was no evidence of Gadolinium enhancement. Endoscopic ventriculostomy and septostomy under neuronavigation were performed. The changed location of the intraventricular cyst on the postoperative MRI confirmed its free-floating character (***Figure 2d***).

Subsequently, medical treatment with antiparasitic therapy (Albendazole) and steroids was started for two weeks. The patient responded well to therapy and could leave the hospital after 10 days. Meanwhile the diagnosis of NC was confirmed by serological tests detecting Taenia solium antigen and antibodies (ELISA) in the CSF and serum.

## Discussion

NC is a brain infection caused by the encysted larval stage (cysticercus) of the pork tapeworm Taenia solium. According to the location in the brain there are two forms: parenchymal and extraparenchymal NC. Compared to the parenchymal form, patients with intraventricular and/or subarachnoid disease have a worse overall outcome with a higher morbidity and mortality [1].

Intraventricular NC occurs as the cysticerci reach the ventricles through the choroid plexus, where they may pass freely or become attached to the ependyma. Intraventricular infection appears to be more frequent than previously thought presenting in up to 30% of patients with NC [2].

CT is more sensitive in detecting calcified lesions but has limited sensitivity for identification of intraventricular cysts [3]. In our patient the intraventricular cyst remained invisible on CT. The calcification represents the nodular calcified stage of NC.

Brain MRI is the modality of choice for the detection of extraparenchymal NC. 3D volumetric T2 weighted sequences have enhanced sensitivity for detection of cysticerci in the ventricles or subarachnoid spaces [4]. The intraventricular form of NC is a frequent cause of intracranial hypertension due to CSF outflow obstruction. Intraventricular cysts may be free floating and cause obstruction at the foramina of Monro, the Sylvian aqueduct, or the fourth ventricle and may lead to a rapid clinical deterioration. Intraventricular cysts are most common in the fourth and third ventricle and are less frequently seen in the lateral ventricles [5].

Diffusion-weighted images typically demonstrate diffusion restriction in the scolex which is visible as an eccentric dot in the cyst [6]. This finding is typically seen in the vesicular and colloidal-vesicular stage of NC. The aqueductal stenosis in our patient was due to the presence of a web, most likely secondary to arachnoiditis. Intraventricular cysts may also become adherent to the ependymal wall of the ventricle and result in ependymitis following cyst involution that may lead to intraventricular compartmentalization and make CSF diversion more problematic [2]. The differential diagnosis of an intraventricular cystic lesion includes neoplastic and infectious lesions but the demonstration of a diffusion restrictive scolex is pathognomonic for intraventricular NC.

## Conclusion

Intraventricular NC is a rare cause of acute obstructive hydrocephalus. With increased international travel and immigration, one should consider NC as a possible etiology. The demonstration of a diffusion restrictive scolex on brain MR imaging is pathognomonic for NC.
